# Association of single nucleotide polymorphisms in the genes *ATM*, *GSTP1*, *SOD2*, *TGFB1*, *XPD* and *XRCC1* with risk of severe erythema after breast conserving radiotherapy

**DOI:** 10.1186/1748-717X-7-65

**Published:** 2012-04-26

**Authors:** Annette Raabe, Katharina Derda, Sebastian Reuther, Silke Szymczak, Kerstin Borgmann, Ulrike Hoeller, Andreas Ziegler, Cordula Petersen, Ekkehard Dikomey

**Affiliations:** 1 Department of Radiotherapy and Radiooncology, Laboratory of Radiobiology & Experimental Radiooncology, University Cancer Center Hamburg, University Medical Center Hamburg-Eppendorf, Martinistr 52, D-20246, Hamburg, Germany; 2 Institute of Medical Biometry and Statistics, University at Lübeck, University Hospital Schleswig-Holstein, Campus Lübeck, Lübeck, Germany; 3 Department of Radiotherapy, Charité University Hospital, Berlin, Germany

**Keywords:** Single nucleotide polymorphisms (SNPs), Erythema, Breast cancer, Radiotherapy

## Abstract

**Purpose:**

To examine the association of polymorphisms in *ATM* (codon 158), *GSTP1* (codon 105), *SOD2* (codon 16), *TGFB1* (position −509), *XPD* (codon 751), and *XRCC1* (codon 399) with the risk of severe erythema after breast conserving radiotherapy.

**Methods and materials:**

Retrospective analysis of 83 breast cancer patients treated with breast conserving radiotherapy. A total dose of 50.4 Gy was administered, applying 1.8 Gy/fraction within 42 days. Erythema was evaluated according to the Radiation Therapy Oncology Group (RTOG) score. DNA was extracted from blood samples and polymorphisms were determined using either the Polymerase Chain Reaction based Restriction-Fragment-Length-Polymorphism (PCR-RFL) technique or Matrix-Assisted-Laser-Desorption/Ionization –Time-Of-Flight-Mass-Spectrometry (MALDI-TOF). Relative excess heterozygosity (REH) was investigated to check compatibility of genotype frequencies with Hardy-Weinberg equilibrium (HWE). In addition, p-values from the standard exact HWE lack of fit test were calculated using 100,000 permutations. HWE analyses were performed using R.

**Results:**

Fifty-six percent (46/83) of all patients developed erythema of grade 2 or 3, with this risk being higher for patients with large breast volume (odds ratio, OR = 2.55, 95% confidence interval, CI: 1.03–6.31, p = 0.041). No significant association between SNPs and risk of erythema was found when all patients were considered. However, in patients with small breast volume the *TGFB1* SNP was associated with erythema (p = 0.028), whereas the SNP in *XPD* showed an association in patients with large breast volume (p = 0.046). A risk score based on all risk alleles was neither significant in all patients nor in patients with small or large breast volume. Risk alleles of most SNPs were different compared to a previously identified risk profile for fibrosis.

**Conclusions:**

The genetic risk profile for erythema appears to be different for patients with small and larger breast volume. This risk profile seems to be specific for erythema as compared to a risk profile for fibrosis.

## Introduction

The treatment of malignant tumours by radiotherapy (RT) is limited by the need to avoid unacceptable normal tissue toxicity. Despite advances in RT-technique, treatment modalities as well as therapeutic strategies, normal tissue damage is still a limiting factor in radiotherapy. In this context, late complications are especially important because they are generally progressive and appear to be associated with a lifelong risk [1]. In contrast, acute normal tissue toxicity is generally a transient phenomenon, with symptoms settling within months after treatment. However, these effects are not less clinically relevant, especially when accelerated fractionation schedules or adjuvant radiochemotherapy treatment are used [2-4] with new substances often increasing normal tissue toxicity, e.g. Cetuximab.

Both acute and late normal tissue effects are known to vary considerably, ranging from negligible to severe, even between patients treated with identical schedules. It has been suggested that these variations in clinical radiosensitivity mainly result from differences in genetically determined radiosensitivity, as only 30% of this variation can be attributed to changes in treatment related parameters [5,6]. In addition to clinical and genetic parameters for radiation response, a number of patient-related confounding factors exist influencing adverse effects definitely, some of which probably have yet to be identified. Based on this background, pronounced scientific interest is currently being directed towards the use of genetic markers such as single nucleotide polymorphisms (SNPs) as parameters for the individual risk of experiencing radiation-induced normal tissue toxicity. Studies on the impact of SNPs are either performed following the candidate gene approach or employing genome-wide association (GWA) analysis.

So far, numerous studies have been performed, but the results obtained are heterogeneous and often conflicting; for reviews, see [7,8]. Even for the C-509 T polymorphism in the transforming growth factor 1 (*TGFB1*) gene, which represents one of the most studied SNPs, there are several reports [9-13] showing that this polymorphism promotes chronic inflammatory and fibrotic reactions, but there are also data suggesting a lack of association or even the opposite effect [14-17].

In addition to the identification of those SNPs which are relevant for normal tissue toxicity, their functional consequences concerning the involved molecular pathways and their mechanisms of action are currently of great scientific interest. A respective consortium addressing this topic has recently been established [18].

The discrepancies in the currently available data may be attributed to the fact that with a frequency of about one SNP every 160 to 180 bp [19-21], the vast majority of SNPs are assumed to have no or a small effect on the respective protein or functional pathway. respectively. Therefore individual genetic characteristics must be determined by the combination of several SNPs each one associated with a small effect. This was indicated by previous studies [7,22] which demonstrated a significant association with normal tissue toxicity only if several - each of one only weakly associated SNPs - were combined to a risk score. Such risk scores can easily be created by adding the number of risk alleles per patients and correlating the resulting numerical value with the severity of the normal tissue toxicity.

It is discussed that a risk profile, based on a combination of SNPs in genes which are involved in relevant pathways may vary for the type of normal tissue toxicity scored [7], i.e. different endpoints are characterized by different mosaic-like displays of certain SNPs.

This concept implies that it might be more informative to analyse a certain combination of SNPs in independent studies rather than to change this combination within different studies.

To date, such analyses have only been performed by Andreassen et al. [10,14,23] using certain combinations of SNPs all including SNPs in *TGFB1SOD2XRCC1XRCC3APEX* and *ATM* genes and studying their association with skin fibrosis in post-mastectomy radiotherapy patients. While the agreement between the first two studies analysing two different cohorts was fairly good [10,14], the third study on a larger cohort of patients [14] failed to confirm the association with radiation induced, indicating that future studies should consider other combination of SNPs [7].

In our studies, we concentrated on six SNPs located in genes (*ATM, GSTP1, SOD2, TGFB1, XPD, XRCC1*) involved either in the induction or repair of DNA double-strand breaks and therefore considered to be of relevance for individual radiosensitivity. In a first report, the association of these SNPs with the risk of late tissue effects was analysed for breast cancer patients treated with breast conserving radiotherapy [22]. No significant association with risk was obtained for any individual SNP, as indicated by *p*-values ranging from 0.064 to 0.643. However, when these SNPs were combined into a risk score, a highly significant association (*p* = 0.0005) was found.

We now investigated in a retrospective study on 83 breast cancer patients the association of these SNPs with the risk of acute tissue toxicity in terms of erythema, with special focus on the relevance of breast size. Erythema grade 2 and more was used as clinical endpoint determined by using the RTOG score. DNA extracted from blood was used to determine the SNP status using either PCR-RFLP or MALDI-TOF. The results imply that fundamental differences exist concerning the risk profile for late and acute tissue toxicity, respectively.

## Material and methods

### Patients

Blood samples were collected from 83 patients with breast cancer (BC) Stage I/II (postmenopausal; mean age of 60.1 at time of treatment (standard deviation, SD: 11.5, range 36–80) who had undergone breast conserving surgery and adjuvant radiotherapy to the breast. This cohort is independent of the one used in the previous study adressing fibrosis [22]. Patients were recruited from the Clinic for Radiotherapy and Radiation Oncology, University Medical Center Hamburg-Eppendorf, and from the Clinic of Radiotherapy, Radiation Oncology and Nuclear Medicine, Vivantes Klinikum Neukölln, Berlin, Germany. Ethical permission as well as informed consent was obtained in advance. Experimental studies were blinded for patient’s identity and clinical performance.

Seventy-seven patients were treated with 1.8 Gy per fraction five times per week and six patients received 25 fractions of 2.0 Gy, amounting to a total dose of 50.4 or 50 Gy, respectively. 67% of patients received a boost of 9 or 10 Gy administered in five fractions of 1.8 or 2 Gy, respectively. 75 patients received hormonotherapy with tamoxifen. At 50 Gy, prior to boost application, erythema of the breast, excluding folds and scars, was evaluated using the RTOG score. Scoring of acute toxicity was undertaken by a single investigator. Four scores were defined: grade 0: no change compared to baseline, grade 1: faint or dull erythema/ epilation/ dry desquamation/ decreased sweating, grade 2: tender or bright erythema, patchy moist desquamation or moderate edema, grade 3: confluent moist desquamation, pitting edema, grade 4: ulceration, hemorrhage, and necrosis. The study was approved by the local ethics committee.

### Genotyping

DNA was extracted from the whole blood of the patients using a genomic extraction kit (Macherey & Nagel, Germany). DNA concentrations were determined using a BioPhotometer (Eppendorf, Germany).

*GSTP1* (codon 105, rs1695), *TGFB1* (position −509, rs1800469), and *XRCC1* (codon 399, rs25487) genotypes were determined using PCR-RFLP. The PCR reaction was carried out using 100 ng of genomic DNA in a total reaction volume of 25 μl using PuReTaq Ready-To-Go PCR beads (Amersham, United Kingdom). Primer pairs (Table 1) were used at a concentration of 10 pmol. For *GSTP1*, cycling conditions were 95°C for 5 min, followed by 35 cycles of 95°C for 30 sec, 60°C for 30 sec and 72°C for 30 sec, with a final extension of 72°C for 5 min. The PCR-product of 176 bp was digested with BsmAI at 55°C for 3 h, forming fragments of 91 bp and 85 bp, which were then resolved on 2% agarose gels. For *TGFB1* and *XRCC1*, cycling conditions were 2 min at 95°C, followed by 35 cycles of 95°C for 45 sec, 60°C for 45 sec and 72°C for 45 sec, or 5 min at 94°C followed by 34 cycles of 94°C for 30 sec, 68°C for 60 sec and 72°C for 60 sec, with a final extension of 72°C for 5 min, yielding PCR-products of 419 bp or 615 bp, respectively. These products were then digested using Bsu36I or MspI to form fragments of 190 bp and 229 bp for *TGFB1*, or 376 bp and 239 bp for *XRCC1*.

**Table 1:** SNP characteristics and population

**Gene**	**rs no.**	**SNP ^a^**	**Codon/ position**	**Change inamino acid**	**Function of gene product**	**Primers**		**Relative excess heterozygosity (95% confidence interval)**	**p-value HWE ^b^**
*ATM*	rs1801516	G/A	1853	Asp > Asn	DNA repair	---	---	0.802 (0.350 – 1.861)	0.842
*GSTP1*	rs1695	A/G	105	Val > Isol	ROS pathway	5′-ACCCCAGGG CTCTATGGGAA-3′	5′-TGAGGGCACA AGAAGCCCCT-3′	1.104 (0.670 – 1.815)	0.924
*SOD2*	rs4880	C/T	16	Ala > Val	ROS pathway	---	---	1.798 (1.130 – 2.849)	0.037
*TGFB1*	rs1800469	C/T	pos.509	–	Pro-fibrotic cytokine	5′-CAGACTTCTAGA GACTGTCAG-3′	5′-GTCACCAGA GAAAGAGGAC-3′	0.993 (0.640 – 1.549)	0.987
*XPD*	rs13181	A/C	751	Lys > Gln	DNA repair	---	---	1.030 (0.640 – 1.656)	0.987
*XRCC1*	rs25487	G/A	399	Arg > Gln	DNA repair	5′-TTGTGCTTTC TCTGTGTCCA-3′	5′-TCCTCCAGCC TTTTCTGATA-3′	0.735 (0.460 – 1.164)	0.424

^a^ major allele / minor allele.

^b^ p-value of the exact Hardy-Weinberg equilibrium lack of fit test.

Genotyping for the polymorphisms in *ATM* (codon 1853, rs1501516), *SOD2* (codon 16, rs4880) and *XPD* (codon 751, rs13181) was performed by Bioglobe (Hamburg, Germany) employing the MassARRAY® system (Sequenom, USA), applying the MassEXTEND® [24] (hME) method and MALDI-TOF mass spectrometry for analyte detection. Reactions were performed according to the standard hME protocol recommended by the system supplier. The protocol generates allele-specific analytes in a primer extension reaction applying the primer directly adjacent to the SNP site. After sample conditioning, a MassARRAY® Analyzer Compact was used for data acquisition, followed by automated data analysis with TYPER® RT software version 3.4. Where necessary, the results were reviewed and the operator was revised. Assay design was successfully performed with platform-specific software for the SNP sequence. Failed reactions/samples were repeated. SNP assays were designed automatically using the MassARRAY platform specific Assay Design software. Additional information was obtained from databases to aid in selecting highly specific primers.

For *ATM*, a second SNP (rs1801673) has been documented in the direct vicinity (next base pair) of the SNP of interest (rs1801516). Therefore, the assay design was modified to allow for the detection of potentially occurring haplotypes, thus ensuring accurate results. Any risk of potential overlap with the secondary SNP was prevented.

In all cases positive controls were included and ambiguous results verified by means of replication.

### Statistical analysis

Relative excess heterozygosity (REH) [25] was determined to check compatibility of genotype frequencies with Hardy-Weinberg equilibrium (HWE). In addition, p-values from the standard exact HWE lack of fit test were calculated using 100,000 permutations. HWE analyses were performed using R.

Patients were divided into two groups with either no or only moderate erythema (grade 0 and 1) or with severe erythema (grade 2 and 3). Analyses were performed using all patients and subgroups stratified by breast volume. Small breast volume was defined as a volume < 750 cm^3^, which is the median volume of all patients. Association between breast volume and risk of erythema was tested using the two-sided asymptotic Cochran-Armitage trend test with odds ratio (OR) and asymptotic 95% confidence intervals (CI) estimated using logistic regression.

Associations between erythema grade and each individual SNP were tested using the two-sided exact Cochran-Armitage trend test. ORs per increase in one allele and exact 95% CIs were estimated using logistic regression. No adjustment was performed for multiple testing. The risk score was calculated by counting the number of risk alleles at five loci. Association with grade of erythema was checked using the two-sided exact Cochran-Armitage trend test assuming an additive effect of both alleles. OR and exact 95%CI were estimated for an increase in one risk allele. Unless stated otherwise, all tests were performed using StatXact with a nominal type I error level of 5%.

## Results

### Acute tissue toxicity

**Figure 1:**
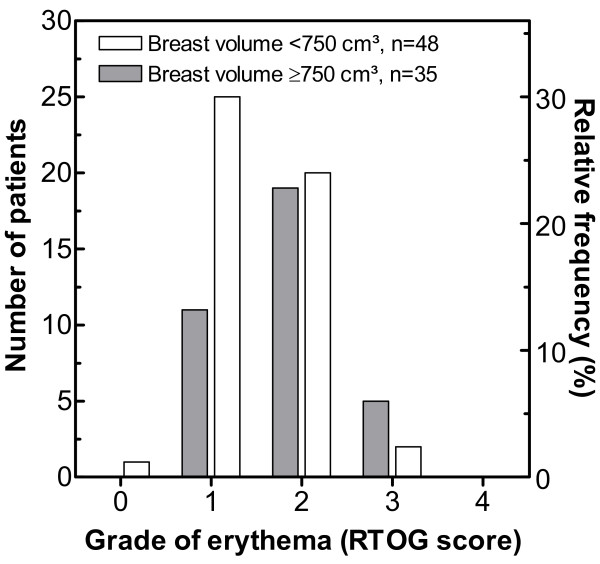
**Incidence of breast erythema in 83 patients treated by breast conserving radiotherapy**. Data are stratified for a breast volume of 750 cm³. Grade of erythema was determined using the RTOG score.

Figure 1 shows the distribution of skin erythema as determined for 83 breast cancer patients using the RTOG score. One patient exhibited no erythema, 36 patients experienced erythema grade 1, 39 grade 2, and 7 patients developed erythema grade 3, while none of the patients showed grade 4. As reported previously [26], a significant association was observed between the risk of erythema and breast volume (OR = 2.55, 95% CI: 1.03–6.31, *p* = 0.041). This is illustrated in Figure 1 showing that 48 patients with a small breast volume had a 46% risk to develop erythema of G2 or 3, in contrast to a 69% risk for 35 patients with large breast volume.

No significant difference was observed in mean age or other individual factors such as smoking habits (data not shown). An analysis using the log-rank test also failed to reveal differences with respect to adjuvant chemo- or hormonal therapy, beam quality or fractionation regime.

### Genotype analysis

DNA extracted from blood samples was used to determine the genotype frequency of the six different polymorphisms (Table 1). In the case of *XPD*-SNP, genotyping failed in one sample, reducing the total number to 82 patients. The obtained genotype frequencies were comparable to those documented for control populations of European descendent (http://www.ncbi.nlm.nih.gov). For *SOD2*, a relative excess heterozygosity of 1.80 (1.13–2.85) was observed, indicating a deviation from HWE (Table 1). This might result from copy number variations, as documented for this locus [27] (see also http://projects.tcag.ca/variation/). To avoid misinterpretation of the data, this SNP was excluded from further analysis.

Table 2 shows the data obtained from the individual SNP association analysis performed for all patients as well as subgroups of patients with small and large breast volumes. Analysing all patients yielded no statistically significant association between erythema and any polymorphism, with p-values ranging between 0.098 and 1 (Table 2, left part). In contrast, a separate analysis of patients with small breast volume revealed a significant allele-dose dependent association for *TGFB1* C-509 T (OR = 3.10, 95% CI: 1.11–10.21, *p* = 0.028). No significant association was found for any of the other four SNPs (Table 2, middle part), but subgroup analysis for patients with larger breast volume yielded a significant association for codon 751 of *XPD* (OR = 3.95, 95% CI: 0.91–22.75, *p* = 0.046). Again, no significant association was seen for the other four SNPs.

**Table 2:** Association of SNPs with erythema for all patients as well as for the subgroups of patients with breast volume < or >750 cm³

			**all patients**							**patients with breast volume <750 cm³**							**patients with breast volume ≥750 cm³**					
			**(n = 83)**							**(n = 48)**							**(n = 35)**					
Gene (codon)	Genotype	aa	n (%)	G0/1^a^	G2/3^a^	OR^b^	95% CI^c^	P^d^	aa	n (%)	G0/1^a^	G2/3^a^	OR^b^	95% CI^c^	P^d^	aa	n (%)	G0/1^a^	G2/3^a^	OR^b^	95% CI^c^	P^d^
ATM	GG		63 (76)	28	35	1				36 (75)	20	16	1				27 (77)	8	19	1	0.19 – 5.68	
(1853)	GA		18 (22)	9	9	1.18	0.45 – 3.25	0.826		11 (23)	6	5	1.43	0.38 – 5.67	0.573		7 (20)	3	4	0.92	0.04 – 32.21	1.000
	AA		2 (2)	0	2	1.38	0.20 – 10.59			1 (2)	0	1	2.05	0.15 – 32.15			1 (3)	0	1	0.84		
GSTP1	AA		37 (45)	19	18	1				21 (44)	12	9	1				16 (46)	7	9	1		
(105)	AG		38 (45)	12	26	1.01	0.49 – 2.09	1.000		21 (44)	10	11	0.98	0.39 – 2.44	1.000		17 (48)	2	15	1.25	0.32 – 5.29	0.772
	GG		8 (10)	6	2	1.02	0.24 – 4.35			6 (12)	4	2	0.96	0.15 – 5.97			2 (6)	2	0	1.56	0.10 – 28.03	
TGFB1	CC		29 (35)	14	15	1				18 (38)	13	5	**1**				11 (31)	1	10	1		
(pos-509)	CT		40 (48)	18	22	1.26	0.65 – 2.50	0.530		24 (50)	12	12	**3.10**	**1.11 –10.21**	**0.028**		16 (46)	6	10	0.36	0.10 – 1.14	0.083
	TT		14 (17)	5	9	1.59	0.42 – 6.24			6 (12)	1	5	**9.58**	**1.23 – 104.30**			8 (23)	4	4	0.13	0.01 – 1.29	
XPD ^e^	GG		34 (42)	19	15	1				20 (43)	12	8	1				14 (40)	7	7	**1**		
(751)	GT		38 (46)	14	24	1.85	0.90 – 4.00	0.098		20 (43)	10	10	1.42	0.59 – 3.59	0.420		18 (51)	4	14	**3.95**	**0.91 – 22.75**	**0.046**
	TT		10 (12)	3	7	3.44	0.81 – 16.01			7 (14)	3	4	2.03	0.34 – 12.89			3 (9)	0	3	**15.62**	**0.84 – 517.40**	
XRCC1	GG		36 (43)	17	19	1				25 (52)	14	11	1				11 (31)	3	8	1		
(399)	GA		33 (40)	13	20	1.02	0.54 – 1.93	1.000		17 (35)	9	8	1.13	0.46 – 2.76	0.840		16 (46)	4	12	0.62	0.20 – 1.84	0.464
	AA		14 (17)	7	7	1.04	0.29 – 3.73			6 (12)	3	3	1.27	0.21 – 7.63			8 (23)	4	4	0.39	0.04 – 3.39	

^a^ Patients with erythema of grade 0 or 1 and grade 2 or 3.

^b^ Odds ratio per allele.

^c^ 95% confidence interval.

^d^ Two-sided p-value from the Cochran-Armitage trend test.

^e^ Failure to genotype one patient.

In addition to the single SNP analysis the association of the combination of all SNPs with erythema was tested. For that the allele associated with an increased risk of erythema was defined as risk allele, which was: A-allele for *ATM* and *XRCC1*, T-allele for *TGFB1* and *XPD* and G-allele for *GSTP1*. However, no significant association with risk of erythema, either for all patients (OR = 1.20; 95% CI: 0.90-1.62; *p* = 0.209), patients with small breast volume (OR = 1.36; CI: 0.94-2.04; *p* = 0.098) or for patients with a large volume (OR = 0.89; CI: 0.52-1.50; *p* = 0.712) was detected for this risk profile.

**Figure 2:**
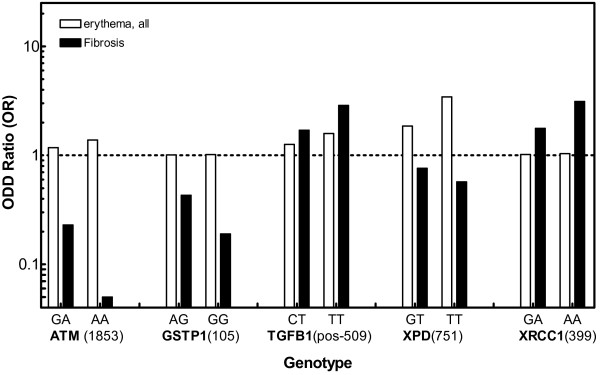
**Association of SNPs in *ATM, GSTP1, TGFB1, XPD* and *XRCC1* with risk of either erythema or fibrosis for breast cancer patients treated by conserving radiotherapy.** Odd ratios (ORs) obtained are plotted vs. the respective gene variants. Data shown for fibrosis are taken from [22].

In Figure 2, Odd ratios (OR) as determined for these five SNPs with respect to risk of erythema (taken from Table 2) are compared with the OR previously determined for risk of fibrosis [22]. Obviously, there is a clear difference between these profiles. In contrast to fibrosis no SNP was found to be associated with an enhanced or reduced risk of erythema (Figure 2, open bars) as indicated by Odd ratios not significantly different from 1,0. When this fibrosis related risk score was applied on the erythema data set no significant association was obtained, (OR = 0.94; CI: 0.71-1.25; *p* = 0.68). These findings emphasizes that the risk of erythema or fibrosis are clearly associated with different SNP profiles.

## Discussion

There is great interest in establishing methods which can be used to predict the individual risk of normal tissue effects after radiotherapy. If these risks were known prior to the onset of therapy, the total dose applied could be reduced in the small proportion of highly sensitive patients and, conversely, radiation dose and possibly the chance to cure could be increased for normal and resistant patients [28,29].

For radiotherapy, the risk of side effects is considered to be determined mostly by treatment-related factors such as dose and dose per fraction, but also to a considerable extent by genetic parameters [5,30]. In this context, SNPs are considered to be the best genetic markers [8].

Following a candidate gene approach, six polymorphisms were selected in genes (*ATMGSTP1TGFB1SOD2XPDXRCC1*) that are known to be associated with the induction or repair of DNA-damage [31-36]. In our first study on breast cancer patients treated by conserving radiotherapy, these SNPs were compared with the risk of severe radiation-induced fibrosis [22]. For each SNP, only a weak association was found, with p-values ranging between 0.064 and 0.643. However, combining all six SNPs into one risk score yielded a highly significant association (*p* = 0.0005).

In this study, we have now analysed the association of the same SNPs with the risk of acute effects as observed after breast conserving radiotherapy. In order to increase the performance of the study, the inclusion criteria were applied strictly for all treatment parameters; i.e. variation in the total dose was negligible, as 50 or 50.4 Gy were applied using only two fractionation regimes (2.0 or 1.8 Gy). Since total dose is considered to be a crucial factor in studying genetic determinants of radiation response [30], the detriment of a relatively small patient collective was counterbalanced by the extremely homogenously performed treatment.

The association between polymorphisms and acute effects was analyzed using the trend test, which reflects the dosage effect of the number of mutant alleles. Due to deviation from the Hardy-Weinberg-equilibrium, one SNP (*SOD2*, codon 16) was excluded from the analysis (Table 1). When the analysis was performed with all patients, none of the five SNPs studied was found to show a significant association with erythema (Table 2). This is in line with most other studies in breast cancer patients (for review see [8]) showing no significant association with acute effects for the vast majority of SNPs. An association has only been found for *GSTP1* codon 105 [37] - which was, however, not observed in our report (Table 2) - as well as for SNPs in *IL12RB2* and in *ABCA1*[38] as well as *CD44, MAD2L2, PTTG1, RAD9A* and *Lig3*[39].

Since a previous study [26] proved breast volume to be of importance for the incidence of radiation induced erythema, and other reports also verify breast size as an important confounding factor in the development of radiation-induced erythema [16,40-43] we performed an additional analysis dichomising the patients according to their breast volume. This analysis yields a significant association of *TGFB1* C-509 T for patients with smaller breast volumes and *XPD* codon 751 for patients with larger breast volume (Table 2). Due to relative small sample sizes we abstained from Bonferoni-correction, following other studies on this issue {[8,44,45] for Review see [8,46]. It cannot be excluded that the observed differences in SNP associations between breast size might be the result of random fluctuations rather than being a reflection of a true difference in the radiobiology of large and small breasts, respectively. Therefore verification studies are needed to test the evolving hypothesis that different pathways and with that different SNPs might be relevant for clinical radiosensitivity according to breast size - and by that possibly to body mass index and metabolic parameters.

For the other four SNPs, no significant association was found (Table 2), even when combined into a risk score. These results indicate that probably due to differences between smaller and larger breasts with respect to micro-milieu and/or metabolism, other parameters and with them other genes are of relevance for the formation of acute effects. As a consequence, more significant associations could potentially have been found in other reports if breast volume had been considered as a relevant confounding factor as done here (Table 2). It has also been shown that the total dose applied must be considered as a relevant confounding factor in a radiogenomic analysis [23,47]. Certainly more of these factors need to be taken into consideration in order to detect the moderate effects caused by a single SNP.

For the five SNPs studied, we also demonstrated that the risk profile obtained for erythema was clearly different from that previously found for fibrosis in breast cancer patients treated by conserving radiotherapy [22]. There are also several reports on breast conserving radiotherapy in which the association between certain SNPs was compared with both acute and late effects. The by far largest study was performed by Chang-Claude and colleagues, analysing the association between polymorphisms in certain DNA repair genes and either acute [48] or late side effects [49]. Although these studies did not always use the same set of polymorphisms, the data obtained confirm that for different clinical endpoints, the risk is determined by different combinations of SNPs. There are numerous reports investigating the impact of mutations in *ATM* on side effects in breast cancer patients. A clear different influence was seen in one study [50], but not in the others [51-53].

The results obtained in the present study indicate that the endpoints of acute and late tissue toxicity are determined by different molecular and cellular pathways, respectively. Therefore, analysing the association of SNPs with both acute and late effects will help us not only to identify the SNPs which might be used as markers of the respective risks, but also to unravel the underlying biological mechanisms.

## Conclusion

In summary, this study demonstrates for the first time that significant associations between a specific SNP and risk of erythema can be identified if breast cancer patients are grouped by their breast volume. The combination of SNPs using risk alleles according to erythema is substantially different from a risk score previously defined for risk of fibrosis. However, these results need to be replicated in an independent and larger study.

## Abbreviations

SNP, Single Nucleotide Polymorphism; RTOG, Radiation Therapy Oncology Group; PCR-RFLP, Polymerase Chain Reaction based Restriction Fragment Length Polymorphism; MALDI-TOF, Matrix-Assisted-Laser-Desorption/Ionization –Time-Of-Flight-Mass-Spectrometry; OR, Odds Ratio.

## Competing interests

The authors declare that they have no competing interest.

## Authors’ contributions

UH collected and analyzed the data, KD and SR performed genotyping procedures. AR reviewed the data and drafted the manuscript. SR, SS and AZ performed statistical procedures. ED and CP revised the manuscript. KB and ED designed the study and revised the final version. All authors read and approved the final manuscript.

## Conflict of interest statement

It is hereby confirmed that all authors disclose all financial and personal relationships with other people or organisations that could inappropriately influence (bias) their work.
